# From the field: a cryptosporidiosis outbreak among veterinary students associated with activities during the lambing period in Norway during 2024

**DOI:** 10.1017/S0950268824001717

**Published:** 2024-12-27

**Authors:** Lise B. N. Hovd, Alejandro Jiménez-Meléndez, Mathilde S. Varegg, Ian D. Woolsey, Ingrid Olstad, Sigurd J. Mathisen, Olav Reksen, Lucy J. Robertson

**Affiliations:** 1 Parasitology, Department of Paraclinical Sciences, Faculty of Veterinary Medicine, Norwegian University of Life Sciences, Ås, Norway; 2 Faculty of Veterinary Medicine, Norwegian University of Life Sciences, Ås, Norway; 3 Herd Health Services, Department of Production Animal Clinical Sciences, Faculty of Veterinary Medicine, Norwegian University of Life Sciences, Ås, Norway

**Keywords:** *Cryptosporidium*, diarrhoea, gastroenteritis, veterinary pathogens, zoonoses

## Abstract

A recent outbreak of cryptosporidiosis (*Cryptosporidium parvum*, subtype IIdA23G1) among veterinary students associated with extracurricular activities concerned with lambs is described from Norway. Although cryptosporidiosis outbreaks among veterinary students have been frequently reported, this is among the first from lamb contact. *Cryptosporidium* oocysts were detected in samples from two students and three lambs. A questionnaire distributed immediately after the outbreak was recognized, identified an assumed attack rate of 50% based on exposure and illness among exposed students (28 of 56), despite most reporting good or very good hygiene measures. Laboratory diagnostics confirmed infection in two of these. The illness lasted over a week in most students (up to 15 days), but contact with health services was negligible. In addition to implementing measures to reduce the likelihood of further such outbreaks among veterinary students, it is recommended that future outbreaks of diarrhoea among ruminants on the farm should be investigated for aetiological agents.

## Key results

- Lamb-contact cryptosporidiosis outbreaks among veterinary students are rarely described.
- We describe such an outbreak from Norway (spring 2024), with *C. paruvm* subtype IIdA23G1.
- Questionnaire responses indicated a 50% attack rate, despite good hygienic measures.
- Although illness mostly lasted over a week, contact with health services was negligible.
- Measures to reduce the recurrence of such outbreaks are discussed.

Outbreaks of cryptosporidiosis among veterinary students have been commonly reported for many years. A recent survey of veterinary students at all ten veterinary schools in the United Kingdom and Ireland reported that the most prevalent self-reported infection was cryptosporidiosis, which was reported by around 15% of the 467 students who completed the survey [1].

In the literature, information on at least seven outbreaks of cryptosporidiosis in veterinary students has been published in the last 20 years (since 2003), one of which was from Norway [2]. Six of these outbreaks were associated with contact with calves (or cattle) and one with foals. Outbreaks of cryptosporidiosis among the public, particularly children, associated with bottle-feeding or close contact with lambs during farm visits, have been reported, especially from the United Kingdom [3], but also from Norway [4]. Although outbreaks of cryptosporidiosis among veterinary students following contact with lambs have not previously been published, some students’ responses in the survey paper [1] mention lambing as being associated with zoonotic infection. Here we describe an outbreak of cryptosporidiosis among a cohort of veterinary students in Norway, participating in a non-compulsory extracurricular activity during the lambing period.

Participation in placements where students assist with lambing and neonatal care of lambs is integral to the education of veterinary students at the Faculty of Veterinary Medicine, at the Norwegian University of Life Sciences (NMBU-VET). In spring 2024, third-year students were offered the possibility to participate in overnight sessions attending the sheep flock held at the university farm, with the main tasks including help with parturition, bottle-feeding of lambs, general care of weak or ill animals, and tube feeding as required. In addition, some cleaning (high-pressure hose and disinfection of stalls etc.) could be performed. The flock consists of breeding ewes of different breeds; in the week before lambing, these included Norwegian White Sheep (*n* = 81), Old Norwegian Short Tail Landrace (*n* = 30) and mixed breeds (*n* = 9). During the 2024 lambing season, 251 lambs were born. Every lambing season, several lambs are taken from their dams to be bottle-fed after receiving colostrum, if the dam cannot provide for all of her lambs (e.g., ewes birthing triplets or quadruplets). The bottle-fed lambs (between 35 and 40; some are fostered by other sheep after a period of bottle feeding), of mixed age and breed, were kept in a separate pen. Ewes with lambs were kept in a common pen. Intermittent diarrhoea was observed in both the bottle-fed lamb pen and lambs in the common pen, and often occurred a couple of days after new individuals were introduced to the pens. Close contact between the bottle-fed lambs and students was frequent, especially during feeding times, and snuggling with the lambs was a regular practice. There is no previous history of diarrhoea in neonatal lambs in the flock, although *Cryptosporidium* infections in sheep (age unknown) at this facility were diagnosed in 2022, but only *Cryptosporidium xiaoi* and *Cryptosporidium hominis* were identified [5]. However, an outbreak of diarrhoea was observed among the goat kids in 2024, a few weeks before the start of lambing, and one employee working with the kids also developed gastrointestinal symptoms. The aetiologies of the diarrhoea among the goat kids and the employee were not investigated. The goat flock was kept in the same building as the sheep, but in different rooms. The same personnel attend to both the flocks and there are no strict hygiene locks between these rooms.

A few days after participating in the lambing activity, several students complained of illness. Faecal samples from students (*N* = 3) were delivered to the parasitology laboratory at NMBU-VET on the students’ own initiative, requesting an examination for *Cryptosporidium.* In addition, samples from five bottle-fed lambs were subsequently delivered. Smears were examined by immunofluorescence antibody (Aqua-Glo™, Waterborne Inc., New Orleans) for *Cryptosporidium* and *Giardia.* Among these samples, three lamb samples were positive for *Cryptosporidium*, two of them with high quantities of oocysts (>100 oocysts per field of view at x250 magnification). Two of the student samples were also positive for *Cryptosporidium*, one with a high number of oocysts. No further faecal samples from either students or lambs were examined. DNA was isolated from positive samples using the DNeasy PowerSoil kit (Qiagen), according to the manufacturer’s instructions, including two cycles of a 60-s bead-beating step. Endpoint polymerase chain reaction (PCR) targeting the *GP60* gene was run on the DNA extract using previously published primers [6], with Fw AL3531 and Rv AL3535 for the first PCR reaction, and Fw AL3532 and Rv AL3534 for the second PCR reaction. Following gel electrophoresis, the amplified DNA was purified and sent for Sanger sequencing in both directions at a commercial facility. The online tool CryptoGenotyper in Galaxy [7] was used to identify the species and subtype of *Cryptosporidium.* All five positive samples were found to contain *C. parvum* subtype IIdA23G1. One representative sequence from a lamb and one from a human sample have been submitted to GenBank (Accession numbers: PQ212612 and PQ212613). This subtype was first described in dairy calves from Spain [8] and the authors of that paper suggested that it may be a subtype adapted to calves as it had not been detected in lambs or goat kids. The same subtype was subsequently reported in *Cryptosporidium* infections in cattle in Sweden [9] and elsewhere in Europe. However, infections in small ruminants with the same *GP60* subtype have since been reported in both Poland [10] and Greece [11]. Human infection with this subtype was first reported in Australia in a 2009 waterborne outbreak [12] and was also the cause of a cryptosporidiosis outbreak among six veterinary students in Italy in 2013, with apparent transmission from two infected foals [11]. In that incident, all six students reported diarrhoea and abdominal symptoms; five students recovered within 1 week and the other student recovered within 16 days [13]. Subsequently, human infections with this subtype have been reported from both Sweden [14, 15], including a foodborne outbreak, and Norway [5].

In our outbreak, an online survey was prepared in Norwegian (English translation provided in the Supplementary Material) and distributed to all students in their third year of study via email immediately after the outbreak was identified. The purpose of the survey was to determine the number of suspected cases among the students and describe symptoms and illness duration, associated risk factors, and contact with health services. Among the 78 students enrolled in their third year of veterinary studies, responses were obtained from 64 students (response rate of 82%). Of these, 56 (87.5%) students had participated in the lambing activities during that spring. Of the eight students who had not, seven reported that they had experienced no sickness during April or May (post lambing). The one student who reported illness during this period and had not participated in the lambing activities, described the illness as fever, typical influenza with a cough and without any overt gastrointestinal symptoms.

**Figure 1.**
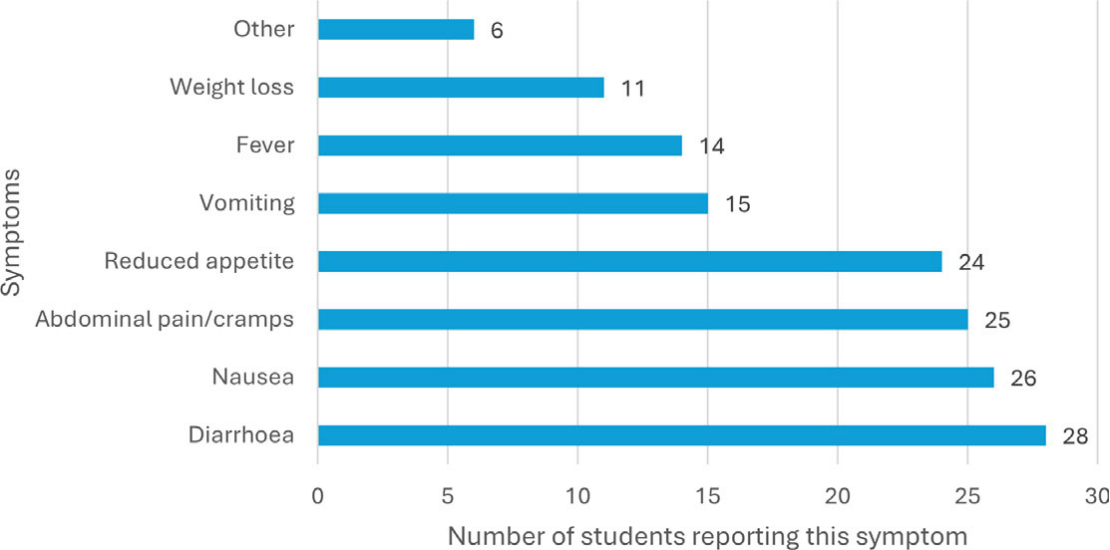
Overview of symptoms reported among veterinary students (*N* = 28) with cryptosporidiosis acquired in association with activities associated with lambing.

Of the 56 students who reported participation in the lambing rotation, 29 reported illness in the period after the lambing, 28 of whom reported primarily diarrhoeal disease, whereas the symptoms of the other student were not consistent with a cryptosporidiosis diagnosis. Of these students, three had provided samples for diagnostic analysis, two of which were positive for *Cryptosporidium.* Thus, 28 of the 56 students who had partaken in lambing were considered likely to have acquired cryptosporidiosis (assumed attack rate of 50%) from the lambs, although the infection was not confirmed by laboratory diagnostics in most students. As the students who are assumed to have acquired cryptosporidiosis participated in at least one, but as many as three, lambing activities on different occasions, determination of the incubation period (between infection and symptoms) is not possible. However, most students providing these data (19 of 28) reported commencement of symptoms between 6 and 7 days after their last lambing activity. One student reported the commencement of symptoms 21 days after the last lambing activity; as the incubation period is generally considered not to exceed 10 days, this may be a reporting error (not all occasions reported) or may reflect a secondary spread. The predominant symptom reported was diarrhoea (28/28, 100%), followed by nausea (26/28, 93%), abdominal pain (25/28, 89%) and a reduced appetite (24/28, 86%); see Figure 1. The majority of students reported being ill for over 1 week, ranging from 2 to 15 days, and with a mean and median duration of 8 days. Over 90% (25/28) of the students with suspected cryptosporidiosis did not contact the health services regarding their illness and mostly self-medicated with non-prescription drugs (painkillers (nine), anti-nausea medication (two), anti-diarrhoea medications (five) and laxatives (one)) and fluids.

Most students who participated in the lambing activities and became unwell (28 with presumed cryptosporidiosis and one with other symptoms) had seen (66%; 19/29) or been told about (17%; 5/29) the lambs having diarrhoea, but the majority had considered that their hygienic measures had either been good or very good (90%, 26/29, for handwashing and 72%, 21/29, for glove use). One student reporting very good hygiene commented on using a disinfection spirit for cleaning their mobile phone.

Coveralls, rubber gloves, soap and warm water are readily available on the farm and instruction in the proper use of personal protective equipment is already implemented. However, following this outbreak, plans for improved preventive measures have already been made for future cohorts of veterinary students to decrease the possibility of another outbreak. Introductory lectures on the lambing activities will place more emphasis on zoonotic diseases and biohazards. Students will be instructed not to bring cell phones and other personal belongings into the lamb enclosures and will be informed that hand disinfectants are not effective against *Cryptosporidium* oocysts. They will be urged to implement hygienic routines immediately after contact with animals and to ensure that they do not spend too long in close contact with lambs, even those lambs without overt symptoms. Reducing the number of students attending each activity should facilitate a better student-to-instructor ratio.

In addition, instigating a sampling routine of ewes and newborn lambs has been recommended to map the infection pressure of *Cryptosporidium* in the flock. This is in consideration of both regarding zoonotic transmission to employees and students, and reducing infection of newborn lambs. In addition, diarrhoeal disease in other animals, particularly goat kids, should be investigated for aetiology. Employees or students who develop symptoms following contact with the small ruminants will be encouraged to seek a timely diagnosis. It is interesting that none of the animal technicians, who handled the lambs daily during this period, reported illness. This may be due to less close contact with the lambs or more thorough hygiene routines; snuggling with lambs was apparently common among the students, but the animal technicians probably did this considerably less frequently. Lack of infection in the staff could also reflect immunity due to previous infections that may not have been reported.

The main limitation of the investigation of this particular study of an apparent outbreak of cryptosporidiosis among veterinary students participating in lambing activities is that faecal samples from only three students were analysed in the laboratory, and these were not necessarily collected during peak oocyst shedding. Thus, the majority of cases were suspected (based on illness and exposure) rather than laboratory-confirmed. In addition, although the questionnaire was distributed in a relatively timely fashion with respect to when the outbreak occurred and the response rate was good, there had still been some time lapse. Furthermore, it was not possible to estimate the incubation period between infection and illness due to most students (15/28) participating in the activity on two or three different occasions.

In conclusion, here we report a recent outbreak of cryptosporidiosis among veterinary students participating in extracurricular lambing activities, where the infection was laboratory-confirmed in only two of the students. Most students believed their hygiene measures to prevent transmission of zoonotic infections were good or very good, and most were aware that the lambs were suffering from diarrhoea. Such outbreaks associated with lambs have been mentioned anecdotally previously, but the majority of cryptosporidiosis outbreaks among veterinary students are usually associated with calf contact. All students assumed to have been infected in this outbreak suffered from diarrhoea, along with a range of other symptoms, largely abdominal, which mostly took over a week – up to 15 days – to resolve. However, most students did not seek healthcare attention, perhaps because they were rapidly made aware by the veterinary faculty that they had cryptosporidiosis and knew that treatment would only be supportive. Low healthcare seeking was also reported in the survey by Furtado et al. [1], who suggest that bravado, as well as rapid recovery, may be a contributing factor. As that survey addresses all zoonotic infections, it is not clear about the relevance to the cryptosporidiosis outbreak described here.

Cryptosporidiosis is a reportable infection in Norway, but these cases will not be included in the reported data due to the lack of contact with the health services and official reportable diagnosis at a medical microbiology laboratory. This demonstrates how data available on cryptosporidiosis in Norway (e.g., at https://msis.no/) is likely to be a significant underestimate, despite the increase in reporting with the implementation of PCR diagnostic tests in medical laboratories [5].

For future cohorts of veterinary students, measures have already been instigated to reduce the likelihood of future outbreaks and the farm itself has been encouraged to explore the *Cryptosporidium* burden in the flock and investigate any future occurrences of diarrhoea among small ruminants and employees working with them.

## Supporting information

Hovd et al. supplementary material Hovd et al. supplementary material

## Data Availability

Representative sequences (one from a lamb sample and one from a human sample) have been submitted to GenBank (Accession Nos. PQ212612 and PQ212613).

## Supplementary material

For supplementary material accompanying this paper visit http://doi.org/10.1017/S0950268824001717. click here to view supplementary material
