# Supplementary material for: From the field: a cryptosporidiosis outbreak among veterinary students associated with activities during the lambing period in Norway during 2024
Source: Epidemiol Infect. 2024 Dec 27;152:e178. doi: 10.1017/S0950268824001717 (PMC11696588; doi:10.1017/S0950268824001717)
Supplement: Hovd et al. supplementary material [file S0950268824001717sup001.docx]

**Mapping possible outbreaks of cryptosporidiosis in veterinary students**

The purpose of this survey is to obtain information a possible outbreak of cryptosporidiosis in veterinary students at NMBU, spring 2024.

Participation in the survey is voluntary. The personal information requested shall only be used to validate information and for further contact if there is uncertainty about the answers.

All data will be anonymized in any publications.

Contact person:

Lucy Robertson, Head of the Parasitology group, VET-NMBU.

[lucy.robertson@nmbu.no](mailto:lucy.robertson@nmbu.no)

**General information**

1. Which student year do you belong to?
   1. Year 20’
   2. Year 21’
   3. Year 22’
   4. Year 23’
2. Did you participate in lambing rotations this spring?
   1. Yes
   2. No

**Questions 3-14 were available only** **to those students that answered NO to question 2 (i.e., they HAD NOT participated in lambing rotations during the spring)**

1. Were you unwell in April or May?
   1. Yes
   2. No

**Questions 4-12 were available only to those students that answered NO to question 2 (i.e., they HAD NOT participated in lambing rotations during the spring) AND answered YES to question 3 (i.e., they had been ill in April or May)**

1. What symptoms have you had?
   1. Diarrhoea
   2. Fever
   3. Vomiting
   4. Stomack pain/cramps
   5. Nausea
   6. Reduced appetite
   7. Weight loss
   8. None of these symptoms
2. When (date) did your symptoms first appear?
3. Have you been in contact with healthcare services due to illness/symptoms?
   1. Yes
   2. No
4. Have you received a diagnosis for your illness? From healthcare or other experts?
   1. Yes
   2. No
5. If yes to Question 7, what diagnosis did you receive?
6. Have you taken any medication for your illness or any symptom-relieving medications?
   This could also include over-the-counter remedies.
   1. Yes
   2. No
7. If yes to Question 9, please list any treatments taken, including over-the-counter remedies?
8. Have you been in contact with other students who have been sick?
   1. Yes, and they had participated in lambing rotations
   2. Yes, but they had not participated in lambing rotations
   3. No
   4. Not sure
9. How many days were you sick/had symptoms?
   1. 1 day
   2. 2 days
   3. 3 days
   4. 4 days
   5. 6 days
   6. 7 days
   7. More than 7 days
10. Do you have anything more to add?
11. If you are willing to answer more questions, please provide your contact information here
    1. Name and email address

**Questions 15-28 were available only to those students that answered YES to question 2 (i.e., they HAD participated in lambing rotations during the spring)**

1. When did you participate in lambing rotations?
   Please provide start and end dates for each individual shift
2. Did you get sick or experience symptoms after the rotations/assisting with lambing?
   1. Yes
   2. No

**Questions 17-27 were available only to those students that answered YES to question 2 (i.e., they HAD participated in lambing rotations during the spring) AND answered YES to question 3 (i.e., they had been ill in April or May)**

1. What symptoms have you had?
   1. Diarrhoea
   2. Fever
   3. Vomiting
   4. Stomack pain/cramps
   5. Nausea
   6. Reduced appetite
   7. Weight loss
   8. None of these symptoms
2. When (date) did your symptoms first appear?
3. How many days were you sick/did you have symptoms?
   1. 1 day
   2. 2 days
   3. 3 days
   4. 4 days
   5. 6 days
   6. 7 days
   7. More than 7 days
4. Have you been in contact with healthcare services due to illness/symptoms?
   1. Yes
   2. No
5. Have you received a diagnosis for your illness? From healthcare or other experts?
   1. Yes
   2. No
6. If yes to Question 21, what diagnosis did you receive?
7. Have you taken any medication for your illness or any symptom-relieving medications?
   This could also include over-the-counter remedies.
   1. Yes
   2. No
8. If yes to Question 23, please list any treatments taken, including over-the-counter remedies?
9. Were you aware whether any of the lambs had diarrhoea while you were on the lambing rotations.
   1. No. I was not aware of diarrhoea among the lambs.
   2. Yes, I observed it directly
   3. Yes, I was informed about diarrhoea among the lambs, but did not observe it directly
10. How would you evaluate your own hygiene measures in relation to the lambing rotations?
    1. Hand wash

i. Poor

ii. Not very good

iii. Neutral

iv. Quite good

- 1. Use of gloves

1. Poor
2. Not very good
3. Neutral
4. Quite good
5. Do you have anything more to add?
6. If you are willing to answer more questions, please provide your contact information here
   1. Name and email address

**FOLLOW-UP QUESTIONNAIRE FOR STUDENTS REPORTING ILLNESS IN ASSOCIATION WITH THE LAMBING ROTATION THAT LASTED FOR MORE THAN 7 DAYS**

1. What symptoms have you had?
   1. Diarrhoea
   2. Fever
   3. Vomiting
   4. Stomack pain/cramps
   5. Nausea
   6. Reduced appetite
   7. Reduced general condition
   8. Weight loss
   9. None of these symptoms
2. When (date) did your symptoms first appear?
3. How many days were you sick/did you have symptoms?
   1. 8 days
   2. 9 days
   3. 10 days
   4. 11 days
   5. 12 days
   6. 14 days
   7. 15 days
   8. 16 days
   9. More than 16 days
4. Describe the general course of the disease. After how many days with symptoms were they most intense? After how many days did you notice recovery?
5. Do you have anything more to add?
6. If you are willing to answer more questions, please provide your contact information here
   1. Name and email address
